# Identifying Predictors of Heart Failure Readmission in Patients From a Statutory Health Insurance Database: Retrospective Machine Learning Study

**DOI:** 10.2196/54994

**Published:** 2024-07-23

**Authors:** Rebecca T Levinson, Cinara Paul, Andreas D Meid, Jobst-Hendrik Schultz, Beate Wild

**Affiliations:** 1 Department of General Internal Medicine and Psychosomatics Heidelberg University Hospital Heidelberg University Heidelberg Germany; 2 Medical Faculty of Heidelberg Internal Medicine IX - Department of Clinical Pharmacology and Pharmacoepidemiology Heidelberg University Hospital, Heidelberg University Heidelberg Germany

**Keywords:** statutory health insurance, readmission, machine learning, heart failure, heart, cardiology, cardiac, hospitalization, insurance, predict, predictive, prediction, predictions, predictor, predictors, all cause

## Abstract

**Background:**

Patients with heart failure (HF) are the most commonly readmitted group of adult patients in Germany. Most patients with HF are readmitted for noncardiovascular reasons. Understanding the relevance of HF management outside the hospital setting is critical to understanding HF and factors that lead to readmission. Application of machine learning (ML) on data from statutory health insurance (SHI) allows the evaluation of large longitudinal data sets representative of the general population to support clinical decision-making.

**Objective:**

This study aims to evaluate the ability of ML methods to predict 1-year all-cause and HF-specific readmission after initial HF-related admission of patients with HF in outpatient SHI data and identify important predictors.

**Methods:**

We identified individuals with HF using outpatient data from 2012 to 2018 from the AOK Baden-Württemberg SHI in Germany. We then trained and applied regression and ML algorithms to predict the first all-cause and HF-specific readmission in the year after the first admission for HF. We fitted a random forest, an elastic net, a stepwise regression, and a logistic regression to predict readmission by using diagnosis codes, drug exposures, demographics (age, sex, nationality, and type of coverage within SHI), degree of rurality for residence, and participation in disease management programs for common chronic conditions (diabetes mellitus type 1 and 2, breast cancer, chronic obstructive pulmonary disease, and coronary heart disease). We then evaluated the predictors of HF readmission according to their importance and direction to predict readmission.

**Results:**

Our final data set consisted of 97,529 individuals with HF, and 78,044 (80%) were readmitted within the observation period. Of the tested modeling approaches, the random forest approach best predicted 1-year all-cause and HF-specific readmission with a C-statistic of 0.68 and 0.69, respectively. Important predictors for 1-year all-cause readmission included prescription of pantoprazole, chronic obstructive pulmonary disease, atherosclerosis, sex, rurality, and participation in disease management programs for type 2 diabetes mellitus and coronary heart disease. Relevant features for HF-specific readmission included a large number of canonical HF comorbidities.

**Conclusions:**

While many of the predictors we identified were known to be relevant comorbidities for HF, we also uncovered several novel associations. Disease management programs have widely been shown to be effective at managing chronic disease; however, our results indicate that in the short term they may be useful for targeting patients with HF with comorbidity at increased risk of readmission. Our results also show that living in a more rural location increases the risk of readmission. Overall, factors beyond comorbid disease were relevant for risk of HF readmission. This finding may impact how outpatient physicians identify and monitor patients at risk of HF readmission.

## Introduction

Patients with heart failure (HF) are the most commonly readmitted group of adult patients in Germany and other Western industrialized countries [1,2]. Nearly two-thirds of patients with HF are readmitted within 1 year [3]. Accounting for ~1%-2% of the annual health care expenditure, with roughly 60% of the spending attributed to inpatient stays, HF poses a major economic burden for health systems, particularly for those who offer universal health coverage [4]. Besides, readmissions increase the risk of complications and mortality in patients with HF [5]. Therefore, understanding the contributors to readmission for identifying patients at risk would be a major step toward both the improvement of patient care and the reduction of costs associated with HF.

Most studies for prediction of HF readmission are based on data from trials and electronic health records introducing a risk for selection bias [6]. Routinely collected data from statutory health insurance (SHI) companies provide large longitudinal data sets representative of the general population. The advantages include reflecting comprehensive and real-life health care provisions for all insured people [7]. Health insurance is mandatory in Germany, with about 90% of the population having SHI [8]. Membership is open to everyone, independent of income, age, or state of health [9].

Outpatient data can provide a different window into the disease state, for example, outpatient data are known to capture a broader spectrum of comorbidity than may be present in inpatient data alone [10]. This may be crucial to the early identification of individuals at risk of readmission for noncardiovascular reasons in this patient group [11]. Furthermore, understanding the relevance of HF management outside the hospital setting is critical to understanding HF and the factors that lead to readmission [12]. To lower costs and ameliorate the patient’s experience, understanding what noninvasive pathways within regular care should be targeted is vital.

To analyze large databases—such as SHI data—machine learning (ML) algorithms are promising methods. ML algorithms can process big data and identify complex patterns while being able to build both linear and nonlinear models for the association between predictor variables and outcomes [13]. ML techniques in cardiovascular research are an emerging field that may offer support in clinical decision-making [14]. ML approaches have successfully been implemented to predict coronary artery disease and atrial fibrillation [15,16]. A recent review concluded that ML algorithms had better discrimination than conventional statistical methods in predicting readmission risk in HF [17]. A recently published study from the Netherlands [18] investigated the predictors of HF-specific readmission using ML on SHI data. However, most readmissions in patients with HF are for noncardiovascular reasons, such as renal failure or pneumonia [19]. To the best of our knowledge, to date, no study exists that applied ML to only outpatient SHI data to predict all-cause readmission in HF.

The aims of this study were (1) to evaluate the use of outpatient SHI data to predict 1-year all-cause (primary end point) and HF-specific (secondary end point) readmission after an initial admission for HF and (2) to identify and rank relevant predictors for readmission. In order to target patients who are at-risk at the earliest possible stage, we included patients with HF who were hospitalized for the first time for HF and thus were just at the presumed start of the “HF readmission circle.”

## Methods

### Study Population

We obtained anonymized data from health insurance claims (from 2012 to 2018) provided by the AOK Baden-Württemberg, a large German SHI with about 4.5 million insured people. In Germany, about 90% of the population receives coverage by SHI, of which the AOK overall company comprises >30% [8]. Within Baden-Württemberg, where the data used in this study originated, AOK comprises 45.5% of the population covered by SHI.

**Figure 1:**
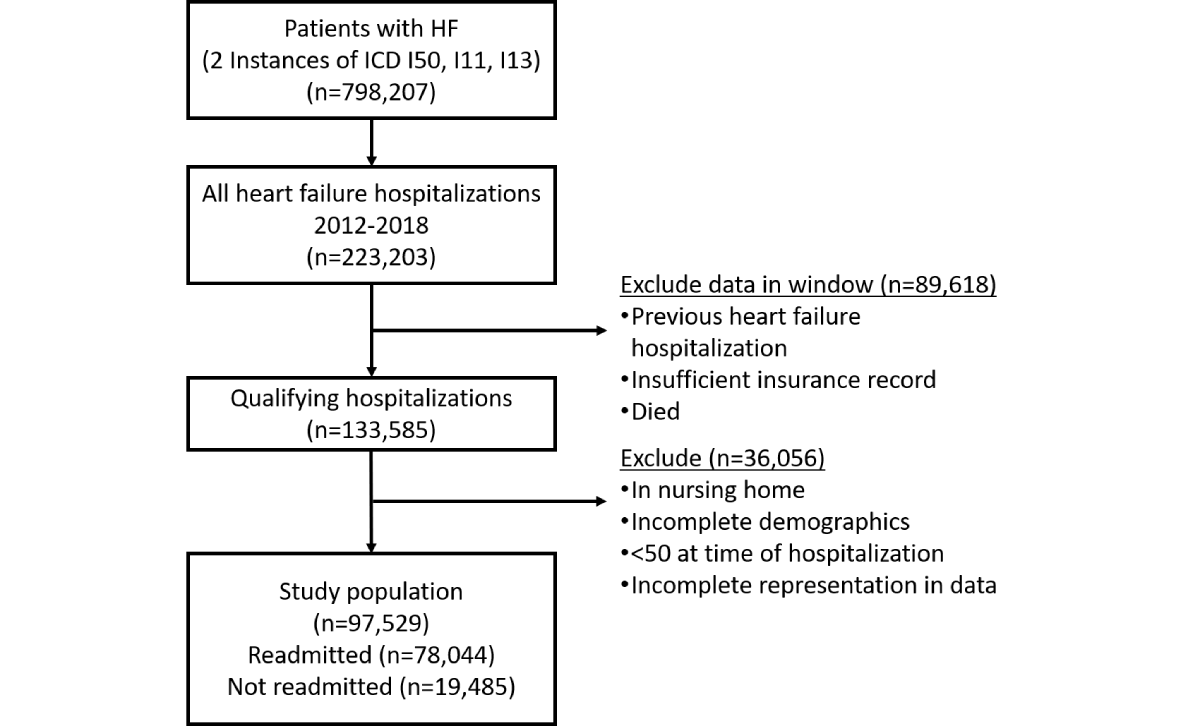
Flowchart for identification of the study population. Patients were identified within statutory health insurance data (2012-2018) from AOK Baden-Württemberg, Germany. *ICD*: *International Classification of Disease*.

We included patients who had HF as documented by 2 or more instances of the *International Classification of Disease, 10th Revision* (*ICD-10*) code I50*, I13*, or I11* on either inpatient or outpatient records and on at least 2 different days. Figure 1 shows the sample selection process. To ensure that patients with 1 readmission were not being compared to those with many, we identified individuals who had their first HF-related admission from 2013 to 2017. All hospital stays were determined from hospital stay data. Hospital stays with shared dates were merged into 1, and at least 3 days were required between the end of the primary HF hospital stay and a potential readmission. To obtain admissions due to HF, *ICD-10* codes documenting reason for inpatient care were mapped to patient stay data. Individuals were required to have a year of record prior to their first HF admission to increase the likelihood of finding the first HF admission for a patient. Individuals were also required to have a year of record after their HF admission, unless they were readmitted. Individuals missing demographics, including date of birth and sex, and those who had insufficient insurance record during the observation period were also excluded. For the remaining population, age at HF diagnosis was calculated and those younger than 50 years at HF diagnosis or who lived in a nursing home were excluded from modeling.

### Study Outcomes

The primary end point of our study was first all-cause readmission within a year after an HF admission. To identify this, all admissions following the first HF admission, deemed the “index admission” in record, were identified. Patients with an all-cause admission 3-365 days after their index admission were considered to have been readmitted. Patients who did not have a readmission within 365 days but were alive and present in the data set on or after the 365-day mark were considered to not have been readmitted within the 1-year window. Patients who died or otherwise withdrew from the insurance scheme prior to the end of the 365 day within and who did not have a readmission were excluded from analysis.

As a secondary end point, we also evaluated the first readmission for HF after the index HF admission. The same methodology as for the primary end point was applied including the time frame of 1 year for readmission. However, readmissions were required to have an *ICD-10* code of I50*, I13*, or I11* attached to them to be considered HF specific.

### Feature Curation and Selection for Prediction Models

To evaluate the role of comorbidities in the prediction of HF rehospitalization, *ICD-10* codes were obtained for all individuals in the study population. Codes were curated to remove entries that did not correspond to an *ICD-10* code and those with dates misentered to be outside the documented time period. Codes after the date of the first hospitalization were also excluded from analysis. *ICD-10* codes were then rolled up into their root code (eg, I25.1 and I25.2 both became I25). Codes on the same day were compiled into 1, and for each individual, the number of unique days each aggregated code appeared was counted. Codes that were part of the Z class of codes, indicating factors relevant to health care use, were also excluded from the analysis. The remaining codes were included as potential features for models. Medications were extracted from prescription medication documentation based on the Anatomical Therapeutic Chemical Classification index (ATC) assigned to each drug. For each ATC number, the number of total packages of a drug was multiplied by defined daily dose to estimate the cumulative in record exposure of an individual to a given drug. Drugs were then filtered to those ATC numbers representing the “C” class of drugs, those affecting the cardiovascular and circulatory system. For each drug, the estimated within data set exposure was included as a potential feature.

Demographic data were also obtained for individuals in the study population. Age, was calculated from date of birth and date of first HF hospitalization, and sex was included as likely relevant to clinical outcomes. Other demographic and demographic-derived variables (described in the following sentences) were included to account for socioeconomic status, professional status, and level of ease of access within the SHI. As we hypothesized that foreigners might have a different relationship with the German insurance system than a German national, a dichotomous variable indicating German nationality was included as a potential predictor. The type of coverage within the SHI was included as a variable with 3 levels, indicating primary holder, family insurance (as the spouse or other dependent family member of the primary insurance holder), or pensioner insurance. To account for a potential disparity in outcomes based on geography (a proxy for both wealth and access to hospitals), data indicating the degree of rurality for each administrative area in Germany were downloaded from the Thuenen Landatlas sponsored by the German Ministry of Food and Agriculture [8,20]. This degree of rurality data was then mapped to the postal codes available in the AOK set, allowing evaluation of degree of rurality in our models. Participation in a disease management program (DMP) focused on diabetes mellitus type 1, diabetes mellitus type 2, breast cancer, chronic obstructive pulmonary disease (COPD), or coronary heart disease prior to the index HF admission was also obtained and included as a binary variable. Measures of cardiac structure or function, such as the output of electrocardiograms, echocardiography, or cardiac imaging, were not available in the data set, and therefore were not included in the prediction models.

### Statistical Analyses

The study population was randomly split with a 70:30 ratio into a training and a testing set for modeling. Using individuals from the study population, 4 models were built for each end point: a logistic regression model, a stepwise regression, an elastic net, and a random forest (RF) model. For each model, potential features with nonmissing data in at least 99% of the training population were included, resulting in 265 features for potential inclusion. As the end point, readmission was unbalanced in the data set, subsampling with 10-fold cross-validation was used to reduce the bias toward predicting only rehospitalization. Elastic net was performed with 5-fold cross-validation, and admission outcomes were weighted based on their prevalence in the data set. Elastic net hyperparameters were tuned using a grid search with an α from 0 to 1 and a λ from 0.0001 to 2. For the RF model, the training set was used for model training and hyperparameter optimization using 3-fold cross-validation. Hyperparameter optimization was performed allowing between 50 and 500 trees, 3 and 20 nodes per tree, and 10 and 50 splits per node. Training was then performed to generate probabilities of readmission for each individual. Within the training set, a cut-point for prediction of readmission was then identified. The training model and cut-point were then evaluated in the testing data. Feature importance indicating the change in model performance due to the exclusion of variables was then generated from the final model. All predictors provided to the elastic net or RF were also included in a logistic regression model and provided in a backwards stepwise regression model. The model then used Akaike information criteria to reduce these features to the minimal set that best predicted HF.

For each modeling approach a C-statistic for model fit was calculated. For models that selected features, important features as determined by mean misclassification error rate through permutation were evaluated. All data management, modeling, and statistical analysis were performed with R (version 3.6.0, 2019-04-06; R Foundation for Statistical Computing) [21]. The packages *tidyverse* [22], *data.table* [23], *ggplot2* [24], *mlr3* [25], *caret* [25,26], and *pROC* [27] were used. For generation of tables summarizing demographics, chi-square tests or Wilcoxon rank sum tests were used as appropriate.

### Ethical Considerations

This work was exempt from specific ethics approval as a secondary analysis of anonymized data (section 303e) [28]. In Germany, analyses of anonymized health insurance data do not require ethics committee approval by law.

## Results

### Population Characteristics

The final sample consisted of 97,529 patients with HF, with a median (IQR) age of 79 (70-85) years and an equal proportion of men (n=49,058, 50.3%) and women (n=48,471, 49.7%). Among them, 78,044 (80%) of the final sample were readmitted to the hospital within the observation period, but only 42,694 (43.2%) were readmitted with HF as one of the primary or secondary diagnoses. Table 1 summarizes baseline characteristics for the final sample and comparisons between those readmitted and not. Overall, readmitted patients were more likely to have pensioners insurance, lived in a more rural location, and had higher rates of outpatient codes for myocardial infarction and COPD. Comparisons between training and testing set are found in Table S1 in Multimedia Appendix 1 and readmission for HF-specific reasons can be found in Table S2 in Multimedia Appendix 1.

**Figure 2:**
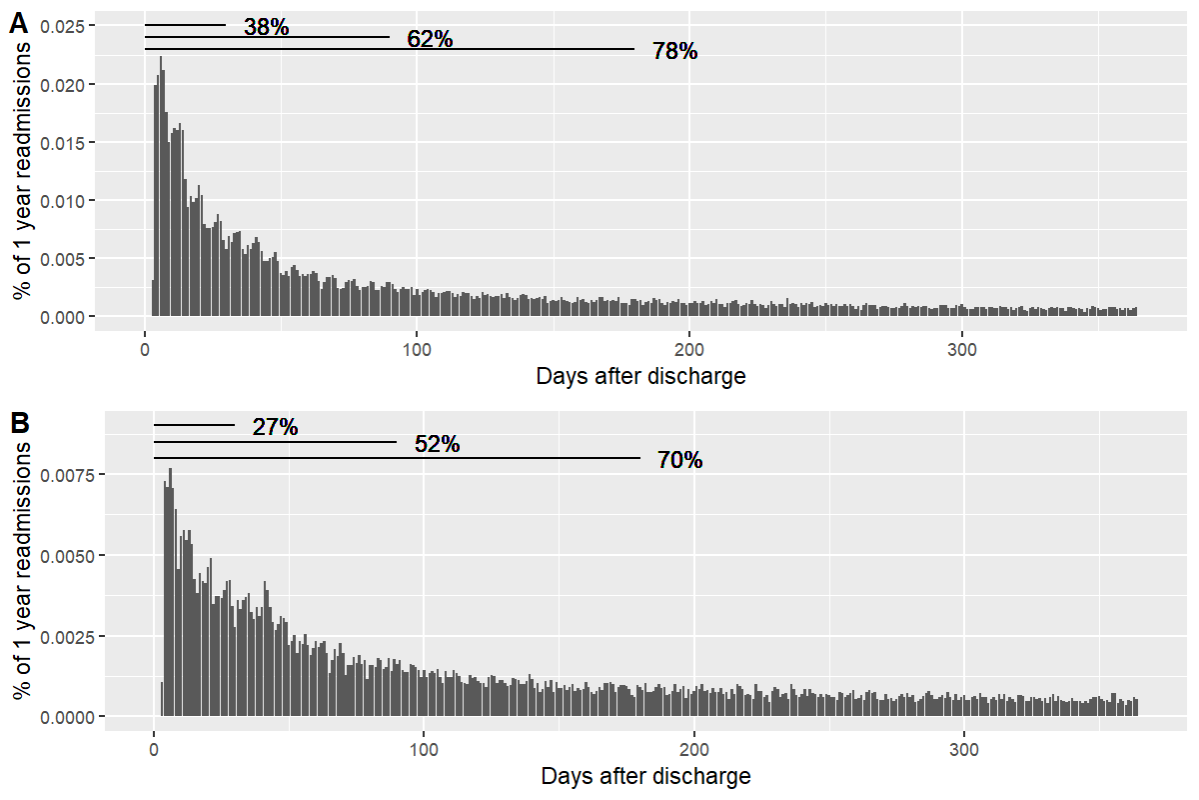
Histogram of time to readmission for readmitted heart failure (HF) patients within the (A) all-cause and (B) HF-specific readmission cohorts. Percentages indicate percentage of the readmitted population for either all-cause or HF-specific readmission.

Individuals who were readmitted within a year after their initial HF hospitalization were often readmitted quickly, with 38% (n=29,747) of readmitted patients returning to the hospital within 30 days, 62% (n=48,628) within 90 days, and 78% (n=60,667) within 180 days (Figure 2A). For the HF-specific readmission end point, although a substantially smaller proportion of the population was readmitted, the trend for time to readmission was similar, with 70% (n=29,896) of readmitted patients readmitted within 180 days (Figure 2B).

**Table 1:** Demographics of the heart failure study population, stratified by all-cause readmission status within the observation period (2012-2018).

		All (N=97,529)	Readmitted (n=78,044)	Not readmitted (n=19,485)	*P* value^a^	
Age (years), median (IQR)		79 (70 to 85)	79 (71 to 85)	79 (70 to 85)	.91	
Sex (male), n (%)		49,058 (50)	40,237 (52)	8821 (45)	<.001	
German national, n (%)		88,249 (90)	70,642 (91)	17,607 (90)	.45	
**Insurance type, n (%)**						<.001
	Primary holder	18,822 (19)	14,725 (19)	4097 (21)		
	Family insurance	2231 (2)	1731 (2)	500 (3)		
	Pensioner’s insurance	76,476 (78)	61,588 (79)	14,888 (76)		
Degree of rurality, median (IQR)		0.06 (–0.52 to 0.53)	0.06 (–0.52 to 0.53)	0.08 (–0.52 to 0.54)	.01	
Hypertension, n (%)		82,198 (84)	65,990 (85)	16,208 (83)	.13	
Atrial fibrillation, n (%)		24,707 (25)	20,340 (26)	4367 (22)	.92	
CAD^b^, n (%)		41,384 (42)	33,942 (43)	7442 (38)	.31	
Myocardial infarction, n (%)		7879 (8)	6612 (8)	1267 (7)	<.001	
Hyperlipidemia, n (%)		51,415 (53)	41,442 (53)	9973 (51)	.84	
Diabetes mellitus type 2, n (%)		41,342 (42)	33,919 (43)	7423 (38)	.71	
COPD^c^, n (%)		20,158 (21)	17,109 (22)	3049 (16)	<.001	

^a^*P* values calculated based on chi-square or Wilcoxon rank sum tests as appropriate.

^b^CAD: coronary artery disease.

^c^COPD: chronic obstructive pulmonary disease.

### Model Performance

The performance of different models for the prediction of first all-cause readmission and the first HF readmission are provided in Table 2. For both, the all-cause and HF-specific readmission end points, the RF model provided the best model fit, with a C-statistic of 0.68 and 0.69, respectively. However, for the HF-specific end point, the elastic net and RF performed very similarly.

**Table 2:** C-statistics for model fit for the 4 applied modeling approaches. Statistics are provided for prediction of 1-year all-cause (primary end point) and heart failure–specific (secondary end point) readmission after an initial admission for heart failure.

	Logistic regression	Stepwise regression	Elastic net	Random forest
All-cause readmission	0.55	0.63	0.65	0.68
Heart failure–specific readmission	0.56	0.65	0.67	0.69

### Predictors of Readmission

As the RF was the best performing model, we evaluated the features with the largest feature importance. The RF feature importance provides a level of importance to the model, but not the direction of the association; therefore, univariate analyses and effect sizes from the elastic net were used to provide additional context.

The most important predictor for the all-cause readmission end point according to the RF model was prescription of pantoprazole (Table 3 and Figure S1 in Multimedia Appendix 1). Other highly relevant features included COPD, sex, diabetes mellitus, atherosclerosis, peripheral vascular disease, age, participation in the coronary heart disease or diabetes DMPs. These predictors included known risk factors for both HF and for general cardiovascular health. In contrast, drugs included in this list tended to be more relevant to general conditions or pain. Degree of rurality was also among the predictors that had an impact on the final model.

For the HF-specific readmission end point, the most important features were the number of times an HF *ICD-10* code had been documented in the medical record prior to the index hospitalization and year of birth (Table 3 and Figure S2 in Multimedia Appendix 1). Other important features included atrial fibrillation, insurance type, chronic back pain, hypertension, degree of rurality, and hyperlipidemia. Overall, the most important features for HF-specific readmission included the majority of the most known and studied HF risk factors and comorbidities. The most important medication for HF-specific readmission was furosemide, a loop diuretic used to treat edema in patients with HF. Sex was significantly less important for the HF-specific model than it is for the all-cause RF. Enrolled in a DMP for diabetes mellitus type 2 or coronary heart disease was also an important predictor in this model.

**Table 3:** Top predictors for 1-year all-cause readmission in patients with heart failure by feature importance from the random forest model.

Feature^a^	Mean misclassification error^b^
A02BC02—pantoprazole	0.05445356
J44—other chronic obstructive pulmonary disease	0.024890419
Sex	0.02361295
E14—diabetes mellitus unspecified	0.015379432
I70—atherosclerosis	0.012226194
I73—other peripheral vascular disease	0.011574568
Age	0.011145327
I25—chronic ischemic heart disease	0.007728684
DM_KHK—DMP^c^ coronary heart disease	0.007057427
DM_DM2—DMP diabetes mellitus type 2	0.005368298
E11—diabetes mellitus type 2	0.005053326
B01AB05—enoxaparin	0.005003516
I10—essential hypertension	0.004787284
R03BB04—tiotropium bromide	0.004478758
B01AA04—phenprocoumon	0.004253736
N18—chronic kidney disease	0.00281131
N19—renal insufficiency not otherwise specified	0.002636976
H02AB06—prednisolone	0.002499561
I35—nonrheumatic aortic valve disorders	0.00248579
M48—other spondylopathies	0.002316437
Degree of rurality	0.002048345
DM_COPD^d^—DMP COPD	0.001441254
A02BC01—omeprazole	0.001138002

^a^Feature name as provided in the data set is listed in the first column, followed by added annotation information, 7-digit codes indicate ATC classifications, and 3-character labels are *ICD-10* codes.

^b^Mean misclassification error represents the change in model score when each variable is randomly permuted.

^c^DMP: disease management program.

^d^COPD: chronic obstructive pulmonary disease.

## Discussion

### Principal Findings

Based on routinely collected health insurance data from >90,000 patients with HF, we have shown that exclusively using outpatient data has clear value for predicting 1-year HF-specific and all-cause readmission.

Interestingly, the 30-day rate of readmission in our analysis was higher than those in the previous studies. We found that 38% (29,747/78,044) of patients were readmitted for any cause, and 27% (11,377/42,694) were readmitted for HF within 30 days. In the same data set, although using a different classification of HF, Ruff et al [2] found that 21% of patients with HF were readmitted for HF within 30 days. It could be that this discrepancy is due to the inclusion of additional years of data with a higher rate of readmission or a difference in study design. However, though high, the rate of readmission seen at 1 year within this population is not implausible, given that others have reported 1-year readmission rates of approximately 67% [3].

The predictive ability of our models is similar to estimates from other retrospective analyses in the real-world data. Van der Galiën et al [18] was able to predict 1-year HF readmission with a C-statistic of 0.71-0.73 including both inpatient and outpatient data in their model. While some models using only inpatient data performed slightly better [29], they lack the ability to make statements about the relevance of health care maintenance outside of the hospital setting to readmission. Other models predicting all-cause readmission using inpatient data were from the United States and considered 30-day admission instead of 1 year. Nonetheless, the predictive performance of our model for 1-year all-cause readmission was slightly better than these, with a C-statistic of 0.68, instead of 0.62 and 0.64 [30,31]. Our best model also outperformed an untargeted analysis in the same data [32], potentially demonstrating the performance gain that can come with careful targeting of both population and model, though the relative contribution of each remains unclear.

Overall, many of the predictors for readmission that we identified as important have previously been mentioned by other studies. Surprisingly, in our data set, pantoprazole was the most important predictor for all-cause readmission. This variable was not mentioned in literature on predictors of readmission in patients with HF before. However, pantoprazole should be probably considered a proxy for overall disease severity. Proton pump inhibitors (PPIs) including pantoprazole are among the most commonly prescribed drugs in the German health care system [33]. PPIs are approved for short term (maximum 12 weeks) use to treat gastrointestinal acid–related disorders [34]. However, studies indicate that PPIs are overprescribed [35], and long-term use of PPIs is associated with increased risk for several adverse health outcomes such as fractures [36] and pneumonia [37]. Noncardiovascular comorbidities are strongly associated with readmission in HF, with pulmonary diseases and bone or joint disorders having the highest proportion among noncardiovascular causes for readmission [38]. Given these findings, exposure to pantoprazole may be a plausible predictor for 1-year all-cause readmission in patients with HF as seen in our data. Nevertheless, these results must be interpreted with caution and should be confirmed in future studies.

Being male was a risk factor for readmission, consistent both with some other HF readmission literature that uses a longer readmission period [39]. Age at which HF occurred was also an important predictor. In univariate and regression models, increasing age was associated with the risk of readmission, an effect that is potentially consistent with previous reported relationships between frailty and HF readmission [40], although this requires further study. We also reported the association of degree of rurality as an important predictor. While other studies have included variables such as distance to the nearest hospital [18], and both the association between rurality with health [41] and rurality with HF prevalence [42], we are, to our knowledge, the first to report this as a relevant predictor for HF readmission. One previous study found that socioeconomically deprived areas had no significant effect on 1-year all-cause readmission in patients with HF using logistic regression [43], but this study did not consider good geographical accessibility of a hospital. Other important predictors such as diabetes, COPD, and coronary disease have been widely and consistently reported in the literature [44-46].

Interestingly, enrolled in a DMP was associated with risk of 1-year readmission in our data. This conflicts with previously published data, also from the AOK Routine Data set Baden-Württemberg, that found that participation in a DMP for diabetes mellitus type 2 was protective in patients with HF against all-cause readmission over an 8-year period [47]. In our analysis, among those not readmitted within 1 year, the rates of participation in DMPs increased with time until readmission. Therefore, we posit that in the short term, participation in DMPs is a marker for chronic disease requiring care and therefore associated with readmission in some patients, but for those who are not quickly readmitted, DMPs can reduce the likelihood of readmission in long term. However, this needs to be confirmed in future studies.

### Limitations

This study has several important limitations. First, we are unaware of any events that occur outside those stated in the data. While we do not expect significant numbers of HF admissions that are undocumented in the data, we cannot be sure whether any occur. Similarly, we have no control over the accuracy of the data set. While we attempted quality control steps to account for clearly impossible data, data points that fell within the plausible spectrum but were incorrect were not adjusted. In addition, due to the nature of health insurance data, no clinical information on HF severity was included. This means that we are able to distinguish the reliability of our predictions for an individual with early versus late stage HF. However, as shown by Desai et al [48], adding electronic health record information to prediction of HF readmission in ML models did not improve model performance. Another limitation is the lack of cardiovascular imaging and measurement. Due to the nature of insurance data, information types that may be relevant in predicting HF readmission, including echocardiography, electrocardiograms, and other imaging data were not available. While other studies have shown these may be relevant for predicting HF, their lack of availability in insurance data is expected. Nevertheless, we recognize that different subsets of patients with HF by ejection fraction may have different sets of predictors that we were unable to evaluate in this study. We also excluded individuals who had HF before 50 years or who lived in nursing facilities. Our conclusions therefore may not be relevant to these populations. One final limitation is the generalizability of our results to the whole German population. Although the AOK Baden-Württemberg covers nearly half of the population in Baden-Württemberg, it is not clear if similar patterns would be apparent within other SHIs or if the characteristics of patients who choose different SHIs would somehow affect this. It is also not clear whether these results are relevant to countries that lack SHIs.

### Conclusions

This study shows that outpatient data from SHI can provide important information for the prediction of all-cause and HF-specific readmission after first admission for HF. It also highlights the relevance of social factors, DMPs, and concerns regularly addressed by primary care physicians in predicting readmission. Future prospective studies are needed to evaluate whether ML models of readmission are accurate in real time and relevant for clinical care.

## Appendix

Multimedia Appendix 1 Population demographics stratified by training or testing set and heart failure (HF)–specific readmission status, as well as information about HF-specific readmission models.

## Abbreviations

ATC: Anatomical Therapeutic Chemical Classification
COPD: chronic obstructive pulmonary disease
DMP: disease management program
HF: heart failure
*ICD-10*: *International Classification of Disease, 10th Revision*
ML: machine learning
PPI: proton pump inhibitor
RF: random forest
SHI: statutory health insurance

## Data Availability

The data sets generated during and analyzed during this study are not publicly available, as they are proprietary of the health insurance company AOK Baden-Württemberg (third-party data), and we are legally not allowed to share these data. Permission to use the data set was granted by the AOK Baden-Württemberg for the specified purpose of readmission analyses within German Innovation Funds project PREMISE according to § 92a (2) Volume V of the Social Insurance Code (§ 92a Abs 2, SGB V—Fünftes Buch Sozialgesetzbuch; grant number 01VSF18019) [49]. Requests to use the data should be addressed to AOK Baden-Württemberg [50]. We hereby confirm that the authors had no special access to the data and that qualified researchers can request access to the data in the same way the authors obtained it.
