# Supplementary material for: Identifying Predictors of Heart Failure Readmission in Patients From a Statutory Health Insurance Database: Retrospective Machine Learning Study
Source: JMIR Cardio. 2024 Jul 23;8:e54994. doi: 10.2196/54994 (PMC11318205; doi:10.2196/54994)

Table S1. Demographics of population divided by training and testing set.

|  |  | All | Training | Testing |  |
| --- | --- | --- | --- | --- | --- |
|  |  | (N=97529) | (N=68258) | (N=29271) | *P* value* |
| Age |  | 79 [70, 85] | 79 [70, 85] | 79 [70, 85] | .68 |
| Male Sex | | 49058 (50 %) | 34406 (50%) | 14619 (50 %) | .32 |
| German National | | 88249 (90 %) | 61771 (90 %) | 26478 (90 %) | .86 |
| Insurance Type | |  |  |  | .92 |
|  | Primary Holder | 18822 (19 %) | 13160 (19 %) | 5662 (19 %) |  |
|  | Family Insurance | 2231 (2 %) | 1558 (2 %) | 673 (2 %) |  |
|  | Pensioner's Insurance | 76476 (78 %) | 53540 (78 %) | 22936 (78 %) |  |
| Degree of Rurality | | 0.06 [-0.52, 0.53] | 0.06 [-0.52, 0.53] | 0.08 [-0.52, 0.53] | .05 |
| Hypertension | | 82198 (84 %) | 57473 (84 %) | 24725 (84 %) | .05 |
| Atrial Fibrillation | | 24707 (25 %) | 17387 (25 %) | 7320 (25 %) | .16 |
| Coronary Artery Disease | | 41384 (42 %) | 29041 (43 %) | 12343 (42 %) | .01 |
| Myocardial Infarction | | 7879 (8 %) | 5519 (8 %) | 2360 (8 %) | .81 |
| Hyperlipidemia | | 51415 (53 %) | 35949 (53 %) | 15466 (53 %) | .70 |
| Diabetes | | 41342 (42 %) | 28895 (42 %) | 12447 (43 %) | .11 |
| Chronic Obstructive Pulmonary Disease | | 20158 (21 %) | 14149 (21 %) | 6009 (21 %) | .05 |

Table S2. Demographics of population for the study population and the subsets of the population with and without HF and specific readmissions.

|  |  | All | Readmitted for HF | Not Readmitted for HF |  |
| --- | --- | --- | --- | --- | --- |
|  |  | (N=97529) | (N=42694) | (N=54842) | *P* value* |
| Age |  | 79 [70, 85] | 80 [73, 86] | 78 [69, 84] | <.001 |
| Male Sex | | 49058 (50 %) | 22057 (52 %) | 27001 (49 %) | <.001 |
| German National | | 88249 (90 %) | 38804 (91 %) | 49441 (90 %) | .001 |
| Insurance Type | |  |  |  | <.001 |
|  | Primary Holder | 18822 (19 %) | 6698 (16 %) | 12121 (22 %) |  |
|  | Family Insurance | 2231 (2 %) | 841 (2 %) | 1385 (3 %) |  |
|  | Pensioner's Insurance | 76476 (78 %) | 35155 (82 %) | 41318 (75 %) |  |
| Degree of Rurality | | 0.06 [-0.52, 0.53] | 0.08 [-0.52, 0.53] | 0.06 [-0.52, 0.53] | .02 |
| Hypertension | | 82198 (84 %) | 36386 (85 %) | 45796 (84 %) | .08 |
| Atrial Fibrillation | | 24707 (25 %) | 12672 (30 %) | 12030 (22 %) | <.001 |
| Coronary Artery Disease | | 41384 (42 %) | 19207 (45 %) | 22171 (40 %) | .006 |
| Myocardial Infarction | | 7879 (8 %) | 3782 (9 %) | 4091 (7 %) | .54 |
| Hyperlipidemia | | 51415 (53 %) | 22361 (52 %) | 29050 (53 %) | .19 |
| Diabetes | | 41342 (42 %) | 19483 (46 %) | 21853 (40 %) | .11 |
| Chronic Obstructive Pulmonary Disease | | 20158 (21 %) | 9657 (23 %) | 10495 (19 %) | .007 |

Table S3. Top predictors for 1-year HF-specific readmission in heart failure patients by feature importance from the random forest model. Feature name as provided in the dataset is listed in the first column, followed by added annotation information after the hyphen. 7-digit codes indicate ATC classifications, and 3-character labels are ICD-10 codes. Mean misclassification error represents the change in model AUC when each variable is randomly permuted.

| Feature | Mean misclassification error |
| --- | --- |
| I50 – Heart Failure | 0.013132 |
| Age | 0.011866 |
| I48 – Atrial Fibrillation | 0.004396 |
| Insurance Type | 0.003415 |
| M54 - Complaints due to back pain | 0.002013 |
| I10 – Essential Hypertension | 0.001086 |
| Degree of Rurality | 0.000956 |
| E78 -Disorders of lipoprotein metabolism and other lipidemias | 0.000954 |
| N95 -Menopausal and other perimenopausal disorders | 0.000949 |
| DM_DM2 - Disease management program Diabetes mellitus type 2 | 0.000734 |
| H52 -Disorders of refraction and accommodation | 0.000649 |
| E11 – Diabetes mellitus Type 2 | 0.000547 |
| J44 – Other chronic obstructive pulmonary disease | 0.000533 |
| I42 - Cardiomyopathy | 0.000448 |
| I25 – Chronic ischemic heart disease | 0.00041 |
| DM_KHK – Disease management program coronary heart disease | 0.000334 |
| C03CA01 - Furosemide | 0.000308 |
| Sex | 0.000305 |
| M01AE01 | 0.000243 |
| M23 -Internal derangement of knee | 0.000188 |
| N18 - Chronic kidney disease | 0.000185 |
| M77 -Other enthesopathies | 0.000155 |
| C09AA05 – Ramipril | 0.000152 |
| M51 -Other damage to intervertebral discs | 0.000146 |
| A02BC02 -Pantopratzol | 0.000146 |
| F32 – Depressive episode | 0.000144 |
| B01AA04 - Phenprocoumon | 0.000122 |
| I73 – Other peripheral vascular disease | 0.000119 |
| M01AB05 - Diclofenac | 0.000119 |
| E66 -Obesity | 0.000115 |
| I35 – Nonrheumatic aortic valve disorders | 0.000113 |
| J06 -Acute upper respiratory infections of multiple and unspecified sites | 0.000107 |

Figure S1. Feature importance from the 1-year all-cause readmission focused random forest. Mean misclassification error represents the change in model score when each variable is randomly permuted.

**Supplemental Figures**


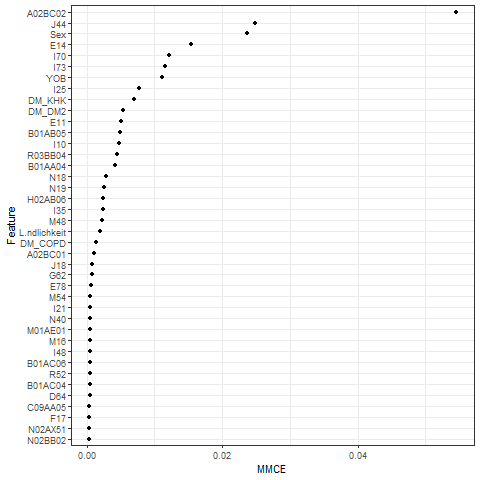


Figure S2. Feature importance from the 1-year HF-specific readmission focused random forest. Mean misclassification error represents the change in model AUC when each variable is randomly permuted.


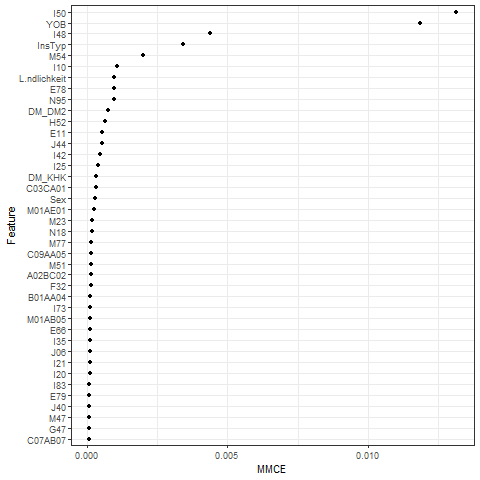

Supplement: Multimedia Appendix 1 [file cardio_v8i1e54994_app1.docx]
